# Luteolin transforms the polarity of bone marrow-derived macrophages to regulate the cytokine storm

**DOI:** 10.1186/s12950-021-00285-5

**Published:** 2021-05-31

**Authors:** Shuxia Wang, Shuhang Xu, Jing Zhou, Li Zhang, Xiaodong Mao, Xiaoming Yao, Chao Liu

**Affiliations:** 1 Clinical Laboratory, Affiliated Hospital of Integrated Traditional Chinese and Western Medicine, Nanjing University of Chinese Medicine, No. 100 Hongshan Road, Nanjing, 210028 China; 2 Jiangsu Province Academy of Traditional Chinese Medicine, Nanjing, 210028 Jiangsu China; 3 Research Center of Endocrine and Metabolic Diseases, Affiliated Hospital of Integrated Traditional Chinese and Western Medicine, Nanjing University of Chinese Medicine, No. 100 Hongshan Road, Nanjing, 210028 China; 4 Department of Pharmaceutical Analysis and Metabolomics, Affiliated Hospital of Integrated Traditional Chinese and Western Medicine, Nanjing University of Chinese Medicine, Nanjing, 210028 Jiangsu China

**Keywords:** Inflammation, Cytokines, Bone marrow-derived macrophage polarisation, Luteolin

## Abstract

**Background:**

Macrophages are indispensable regulators of inflammatory responses. Macrophage polarisation and their secreted inflammatory factors have an association with the outcome of inflammation. Luteolin, a flavonoid abundant in plants, has anti-inflammatory activity, but whether luteolin can manipulate M1/M2 polarisation of bone marrow-derived macrophages (BMDMs) to suppress inflammation is still unclear. This study aimed to observe the effects of luteolin on the polarity of BMDMs derived from C57BL/6 mice and the expression of inflammatory factors, to explore the mechanism by which luteolin regulates the BMDM polarity.

**Methods:**

M1-polarised BMDMs were induced by lipopolysaccharide (LPS) + interferon (IFN)-γ and M2-polarisation were stimulated with interleukin (IL)-4. BMDM morphology and phagocytosis were observed by laser confocal microscopy; levels of BMDM differentiation and cluster of differentiation (CD)11c or CD206 on the membrane surface were assessed by flow cytometry (FCM); mRNA and protein levels of M1/M2-type inflammatory factors were performed by qPCR and ELISA, respectively; and the expression of p-STAT1 and p-STAT6 protein pathways was detected by Western-blotting.

**Results:**

The isolated mouse bone marrow cells were successfully differentiated into BMDMs, LPS + IFN-γ induced BMDM M1-phenotype polarisation, and IL-4 induced M2-phenotype polarisation. After M1-polarised BMDMs were treated with luteolin, the phagocytosis of M1-polarized BMDMs was reduced, and the M1-type pro-inflammatory factors including IL-6, tumour necrosis factor (TNF)-α, inducible nitric oxide synthase (iNOS), and CD86 were downregulated while the M2-type anti-inflammatory factors including IL-10, IL-13, found in inflammatory zone (FIZZ)1, Arginase (Arg)1 and CD206 were upregulated. Additionally, the expression of M1-type surface marker CD11c decreased. Nevertheless, the M2-type marker CD206 increased; and the levels of inflammatory signalling proteins phosphorylated signal transducer and activator of transcription (p-STAT)1 and p-STAT6 were attenuated and enhanced, respectively.

**Conclusions:**

Our study suggests that luteolin may transform BMDM polarity through p-STAT1/6 to regulate the expression of inflammatory mediators, thereby inhibiting inflammation. Naturally occurring luteolin holds promise as an anti-inflammatory and immunomodulatory agent.

## Background

Inflammation is the immune system’s response to invading pathogens, but aberrant inflammatory responses lead to a “cytokine storm” that worsens a patient’s condition [1]. Mounting evidence has shown that continuous and/or repeated inflammatory stimuli can also induce tumours [2]. Therefore, the inflammatory response is a double-edged sword. If immune cells and pro-inflammatory cytokines are overproduced, cytokine cascades occur, called a “cytokine storm” or “inflammatory storm”, leading to sepsis, acute respiratory distress syndrome (ARDS) and even multiple-organ failure (MOF) [3]. It is well known that pathogenic agents such as viral or bacterial infections incur the pathological process of sepsis, which is characterised by an overwhelming generation of pro-inflammatory cytokines. Recently, the global coronavirus disease 2019 (COVID-19) pandemic due to severe acute respiratory syndrome coronavirus 2 (SARS-CoV-2) is also associated with macrophage hyperpolarisation that elicits “cytokine storms” and viral sepsis [4]. Thus, the immunomodulatory therapy for inflammation is crucial for maintaining homeostasis [5].

Macrophages have been identified as critical effector cells in the inflammatory/immune response and can be activated by pathogenic agents or inflammatory mediators to secrete various inflammatory factors. Meanwhile, heterogeneity and plasticity are hallmarks of macrophages, that is, M1-polarised (pro-inflammatory) macrophages and M2-polarised (anti-inflammatory) macrophages [6, 7]. Pathogen infection can polarise macrophages to the M1-phenotype, resulting in the production of high levels of pro-inflammatory cytokines such as interleukin (IL)-6 and tumour necrosis factor (TNF)-α, or effector molecules inducible nitric oxide synthase (iNOS) and surface markers cluster of differentiation (CD)11c or CD86, exert a pro-inflammatory effect and defense against pathogens. Conversely, IL-4 or transforming growth factor (TGF)-β induced M2-phenotype macrophages mainly express anti-inflammatory cytokines IL-10, IL-13, found in inflammatory zone (FIZZ)1, effector molecule Arginase (Arg) 1 and surface marker CD206, which contribute to hinder inflammation [8]. Normally, M1/M2 polarisation of macrophages maintains a dynamic equilibrium. When irritated by virulent bacteria or viruses or overmuch inflammatory molecules, this balance is disrupted, and the macrophages are excessive M1-polarised and generate redundant inflammatory factors, leading to systemic inflammatory response syndrome (SIRS, namely sepsis) and MOF [9, 10]. Therefore, it is particularly vital to skew the macrophage polarisation and avoid excessive M1-polarisation, thus reducing the inflammatory response and promoting tissue remodeling.

At present, most anti-inflammatory agents are glucocorticoids, antibiotics or antivirals. Hormones not only cause immunosuppression, but also induce secondary infections and prolong the disease course or other side effects. Antibiotics or antiviral drugs only kill the pathogen, and that antibiotics lyse the bacteria while killing the bacteria, so releasing more toxins to induce “inflammatory storms”, further exacerbating the inflammatory response and promoting the development of sepsis. In this regard, natural anti-inflammatory immune drugs extracted from plants have multi-effect regulation and less toxic, and especially have obvious benefits in suppressing inflammation. Therefore, it is urgent to seek natural compounds with high efficiency and low toxicity as anti-inflammatory immune agents.

Luteolin (Lut) is a flavonoid, mainly exists in fruits, vegetables and Chinese herbs, which have anti-hyperlipidemic [11], antitumour [12], anti-inflammatory and immunoregulatory activities [13]. Studies have shown that luteolin also has antiviral effects against influenza A virus or dengue virus by interfering with coat protein [14, 15]. Recently research indicated that active ingredients of Chinese medicines, including luteolin and quercetin, could manage COVID-19 by targeting on ACE-2 and 3CL proteins and dampen inflammatory mediators without side effects, and they have achieved significant clinical efficacies [16, 17]. Our previous investigation found that luteolin can regulate the expression of inflammatory factors in macrophages and play an anti-inflammatory role [18], but whether luteolin can transform the polarisation of BMDMs and its molecular mechanism is still unknown. In this research, bone marrow cells isolated from C57BL/6 mice were induced to differentiate into BMDMs for investigating the effects of luteolin on M1/2 polarisation of BMDMs and the expression of inflammatory factors so as to explore the underlying mechanism. Our present findings preliminarily provide luteolin could be a future perspective for the natural anti-inflammatory agent in prevention and treatment of sepsis.

## Methods

### Mice

Six-week-old C57BL/6 mice (weighing 18–22 g) were provided by the Animal Experimental Center of the Affiliated Hospital of Integrated Traditional Chinese and Western medicine, Nanjing University of Chinese medicine (Nanjing, China). Mice were maintained under specific pathogen-free conditions and in accordance with protocols of the Guide for the Care and Use of Laboratory Animals (National Institutes of Health and approved by institutional committee (Ethics Number: AEWC-20181019-53).

### Isolation and culture of BMDMs

C57BL/6 mice were sacrificed by cervical dislocation, and dissected under immersion in 75% ethanol (v/v). The epiphysis was cut after the tibia and femur were separated, and the bone marrow cavity was rinsed with sterile phosphate-buffered saline (PBS) until the bone became white. The bone marrow washing solution was filtered through a 70 μm mesh and transferred to a 50 mL centrifuge tube for cell collection. After lysis of red blood cells, bone marrow stem cells were resuspended in Dulbecco’s modified Eagle’s medium (Gibco, Wellesley Hills, MA, USA) containing 10% foetal bovine serum (AusGeneX, Molendinar, Australia), 100 units/mL penicillin and 100 mg/mL streptomycin and 20 ng/mL macrophage colony-stimulating factor (M-CSF) on 10-cm petri dishes, and renewed the medium every other day. After 7 days of incubation, cells were labeled with F4/80-PE fluorescently conjugated antibodies (1.0 μL; eBioscience, San Diego, CA, USA) to identify the differentiation degree of mature BMDMs by FCM (Millipore, Burlington, MA, USA).

### Cell viability assay

BMDMs were plated in 96-well plates at a density of 2 × 10^4^ cells/well in 200 μL medium to reach confluence overnight. After that, the cells were exposed to LPS (20 ng/mL; Sigma, St. Louis, MO, USA) plus IFN-γ (10 ng/mL; PeproTech, Rocky Hill, NJ, USA) or IL-4 (20 ng/mL; Pepro Tech, USA), and LPS plus IFN-γ-primed cells were administered with luteolin (Sigma, USA) at 2.5 and 5.0 μM for 24 h. Following treatment, cells were treated with 20 μL MTT solution (5 mg/mL; Sigma, MO. USA). After 4 h, the culture medium was removed, and the crystals were dissolved in 150 μL dimethyl sulfoxide per well. The absorption values were measured at 570 nm.

### The morphology of polarised BMDMs

BMDMs in logarithmic stage were cultivated in 6-well plates and incubated overnight, followed by treatment with LPS (20 ng/mL) plus IFN-γ (10 ng/mL) or IL-4 (20 ng/mL). Simultaneously, the cells stimulated with LPS plus IFN-γ were co-cultured with specified concentrations of luteolin for 24 h. Cell morphology was observed under an inverted microscope (Olympus, Tokyo, Japan). Cells and culture supernatants were collected for subsequent mRNA and protein analyses.

### Pathogen phagocytosis assay

Briefly, BMDMs were seeded in 24-well plates at a density of 2 × 10^5^ cells/well and exposed to LPS plus IFN-γ or IL-4 respectively, following luteolin stimuli for 24 h. Subsequently, macrophages were incubated with Gram-positive bacteria or fungi (*Candida albicans*) which have been heat-inactivated at 70 °C for 30 min (the ratio of cells to bacteria is approximately 1:20). 2 h later, cells were washed three times with chilled (4 °C) PBS and stained with Wright-Giemsa Stain (Baso, Zhuhai, China). Phagocytosis of pathogens was visually assessed using an microscope.

### Quantitative real-time PCR (qPCR)

BMDMs were treated as previously described, then cells were collected and total RNA was extracted using Trizol (Ambion Life Technology, Waltham, MA, USA) and reverse-transcribed into cDNA. With GAPDH as the internal reference, qPCR was carried out using SYBR Green Master Mix (Toyobo, Toko, Japan) accoding to primer sequence with Quant studio DX real-time quantitative PCR employing biosystems (Life Technologies, Carlsbad, CA, USA). Relative gene expression was calculated using the 2^−ΔΔct^ comparative method. Primer sequences were obtained from Generay Biotech Co. Ltd. (Shanghai, China) and are listed in Table 1.

**Table 1:** Primers for quantitative real-time PCR

Genes	Forward (5′-3′)	Reverse (5′-3′)
**CD86**	TCAATGGGACTGCATATCTGCC	GCCAAAATACTACCAGCTCACT
**iNOS**	CAGAGGACCCAGAGACAAGC	TGCTGAAACATTTCCTGTGC
**IL-6**	TAGTCCTTCCTACCCCAATTTC	TTGGTCCTTAGCCACTCCTTC
**TNF-α**	CCTCCCTCTCATCAGTTCTA	ACTTGGTGGTTTGCTACGAC
**Arg1**	CAGAAGAATGGAAGAGTCAG	CAGATATGCAGGGAGTC
**CD206**	CAGGTGTGGGCTCAGGTAGT	TGTGGTGAGCTGAAAGGTGA
**FIZZ1**	GCTACTGGGTGTGCTTGTG	CTGGGTTCTCCACCTCTTC
**IL-13**	GGGGAGTCTGGTCTTGTGTGA	CTCTTGCTTGCCTTGGTGGTC
**IL-10**	CTTACTGACTGGCATGAGGATCA	GCAGCTCTAGGAGCATGTGG
**GAPDH**	TGAAGCAGGCATCTGAGGG	CGAAGGTGGAAGAGTGGGAG

### ELISA for cytokines secretion

BMDMs in exponential phase were inoculated in a 6-well plate overnight. Successively, the drugs induced cell polarisation and combined with different concentrations of luteolin for 24 h. The levels of cytokines in the supernatants were performed according to commercial ELISA kits (eBioscience, USA) instructions. Logistic fitting-curve for two of the four parameters was used to calculate the concentration of cytokines.

### FCM staining of BMDM surface markers

Totally, 5 × 10^5^ BMDMs were resuspended in 100 μL PBS and then incubated with 0.5 μL anti-mouse CD16/32 blocking antibody (BioLegend, San Diego, CA, USA) in an ice bath for 20 min to avoid nonspecific binding. Subsequently, cells were stained with anti-mouse FITC-CD11c (0.5 μL; BioLegend, USA) or APC-CD206 (10 μL; BioLegend, USA) and protected from exposure to light for 30 min at room temperature. After washing with PBS, the cells were fixed with paraformaldehyde (0.5 mL) at 4 °C and the mean fluorescence intensity (MFI) of membrane surface antigens CD11c or CD206 was analysed by FCM.

### Protein extraction and immunoblotting

Cells were collected and total protein was extracted for protein quantification by BCA. After 20 μg protein was subjected to SDS-PAGE and transferred to polyvinylidene fluoride membranes (millipore, USA), the corresponding primary antibodies against phosphorylated signal transducer and activator of transcription (p-STAT)1-tyr^701^, p-STAT6-tyr^641^ (Cell Signaling Technology, Danvers, MA, USA) and β-actin (Sigma, USA) were applied at 4 °C overnight. Then the membranes were washed and incubated with HRP-conjugated secondary antibodies with shaking at room temperature for 30 min. Immunoreactive proteins were exposed and developed using enhanced chemiluminescence (Beyotime, China), and β-actin was used as an internal reference to calculate the relative expression of proteins.

### Statistical analysis

The experimental data are presented as mean ± SD. One-way analysis of variance (ANOVA) followed by Tukey’s post-hoc test was used for the multiple comparisons. Analysis was performed using the GraphPad Prism software (Sversion 6.0; an Diego, CA, USA). Statistical significance was set at *P* < 0.05.

## Results

### Mouse bone marrow cells differentiate into BMDMs

**Fig. 1:**
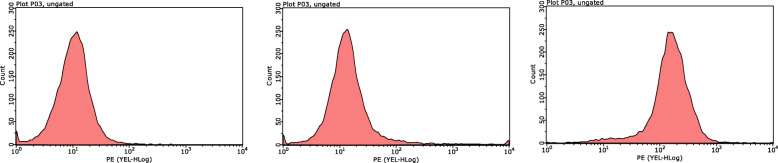
The differentiation proportion of BMDM. FCM detected the surface marker F4/80 of BMDMs after induction for 7 days, and the positive rate was 92.71%

The isolated mouse bone marrow cells were induced by M-CSF for 7 days, and FCM was used to detect the specific marker F4/80 in mouse macrophages. The results showed that the purity of the differentiated macrophages reached 92.71%, indicating that the mouse BMDMs were successfully cultured and could be used in subsequent experiments (Fig. 1).

### Effect of luteolin on BMDM viability

**Fig. 2:**
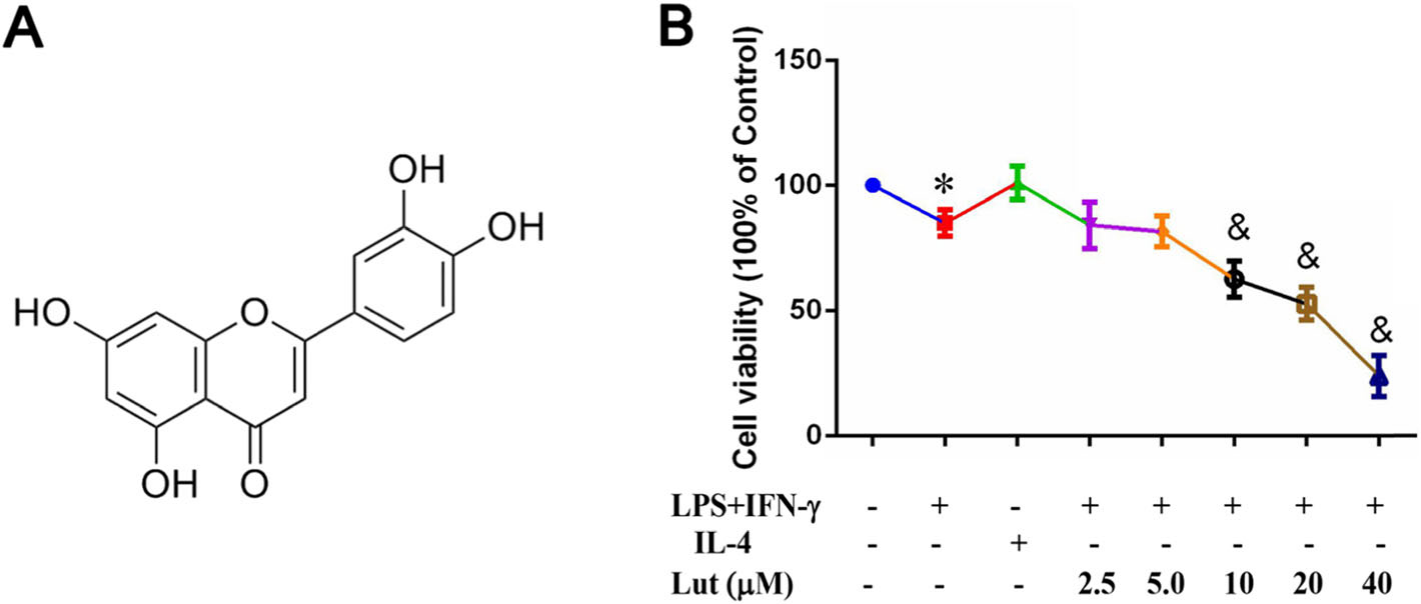
Effect of luteolin on the viability of LPS + IFN-γ-primed BMDMs. **a** Structure of luteolin. **b** BMDMs were primed with LPS + IFN-γ and contributed with indicated doses of luteolin for 24 h, and then the cell viability was assessed by MTT assay. Data represent the mean ± SD of three independent experiments performed in triplicate. Different symbols indicate a significant difference according to ANOVA and Tukey’s test. *P < 0.05 vs. control group (non-treated control); ^&^*P* < 0.05 vs. LPS + IFN-γ-treated group

The non-cytotoxic doses of luteolin were evaluated via MTT assay to exclude the contribution of anti-inflammatory potential of luteolin. Luteolin exhibited no significant impact on the LPS + IFN-γ-primed BMDM proliferation at concentrations up to 5.0 μM at 24 h (Fig. 2b). Therefore, non-cytotoxic concentrations were chosen to assess the bioactivity of luteolin.

### Morphology of polarised BMDMs

**Fig. 3:**
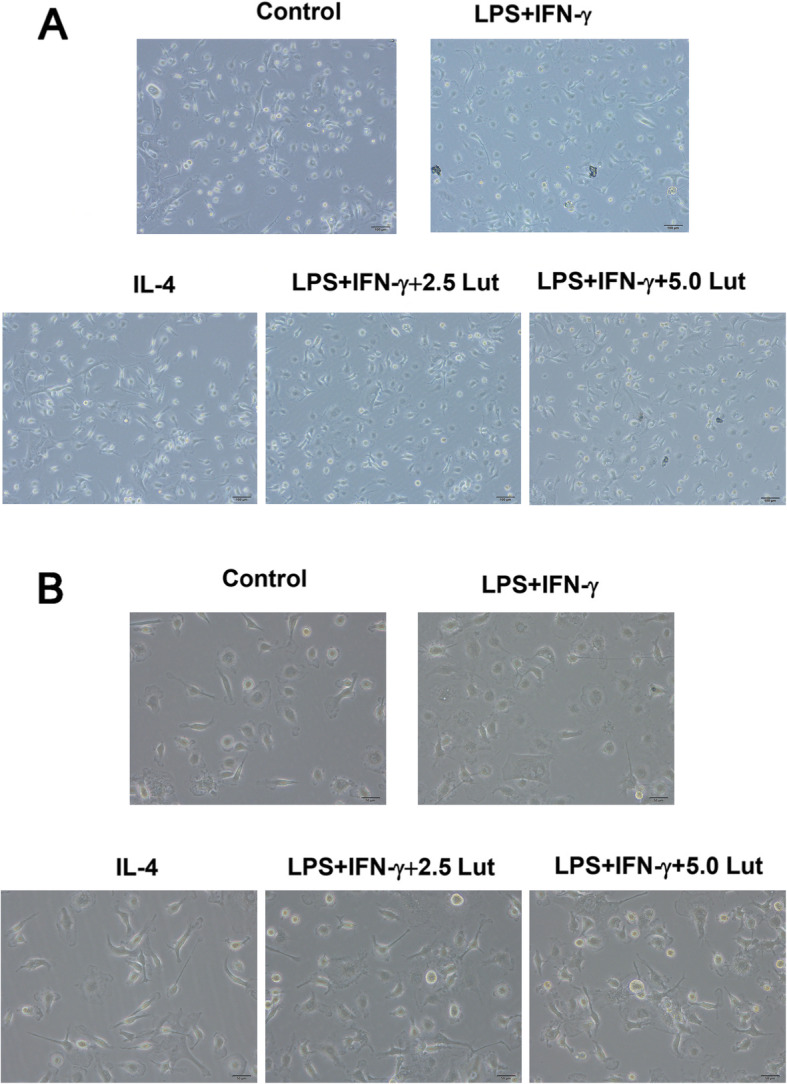
The morphology of polarised BMDMs (**a**. 200×; **b**. 400×). BMDMs were polarised with LPS + IFN-γ or IL-4, simultaneously, BMDMs exposed to LPS + IFN-γ were administrated with luteolin for 24 h. Micrographs of BMDMs were observed using a bright-field Olympus imaging system

Microscopically, BMDMs showed typical macrophage morphology, such as round, oval, or irregular shapes, with pseudopods and adherent growth (Fig. 3 Control group). After being induced into M1-phenotype by LPS plus IFN-γ, the BMDMs presented an oval “fried egg” appearance and pseudopodia extension (Fig. 3 LPS + IFN-γ-treated group), while the M2-type BMDMs induced by IL-4 were round with a plump cytoplasm, accompanied by short pseudopodia (Fig. 3 IL-4-treated group). After various doses of luteolin contribution, M1 cells contracted slightly, and pseudopodia became shorter (Fig. 3 LPS + IFN-γ-combined with 2.5/5.0Lut-treated groups).

### Effect of luteolin on phagocytosis in polarised BMDMs

**Fig. 4:**
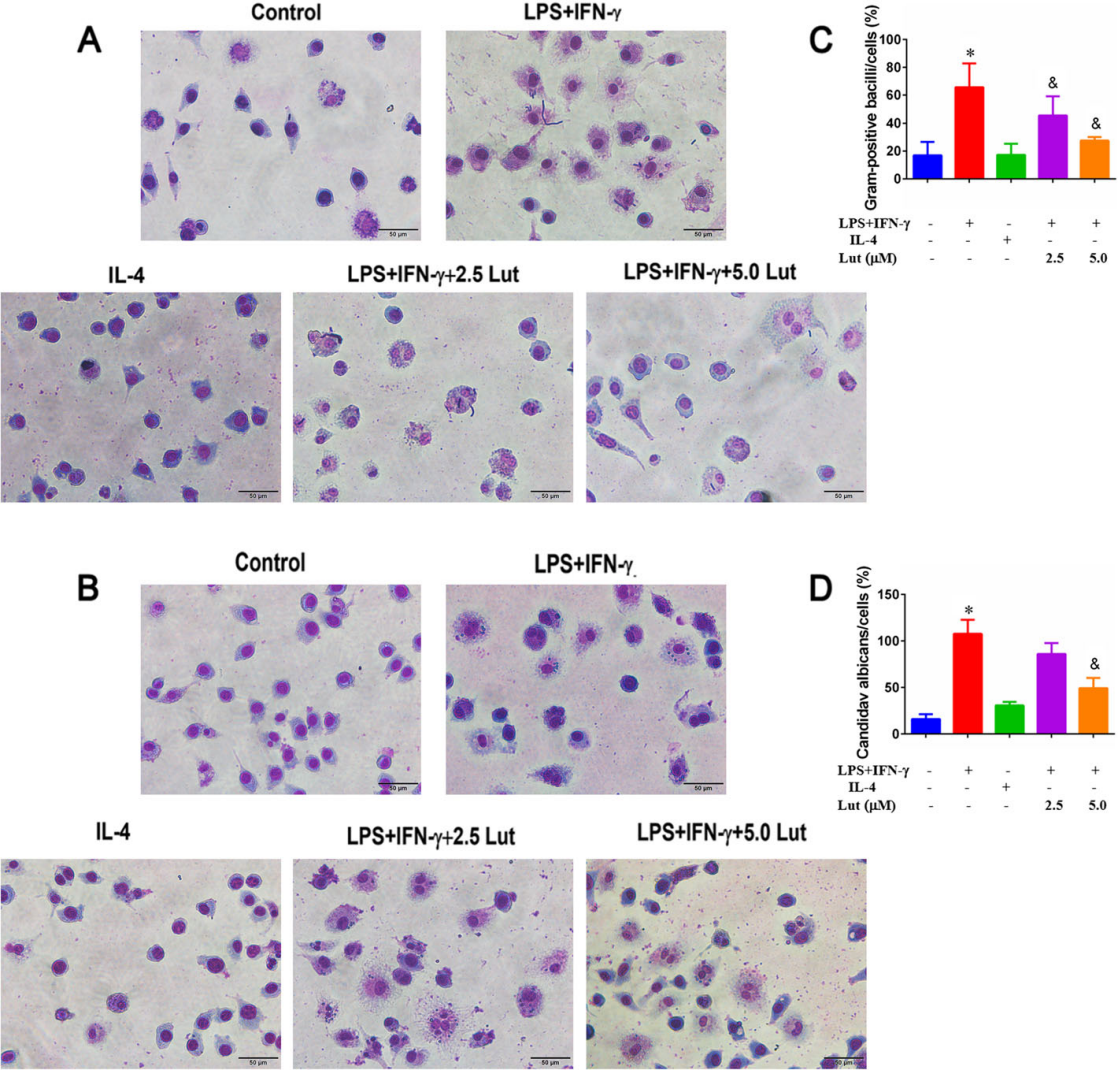
Effect of luteolin on the phagocytic activity in polarised BMDMs. The BMDMs that have phagocytosed Gram-positive bacilli (**a**) or *Candida albicans* (**b**) are stained with Wright-Giemsa Stain to visualize in the field. Phagocytosis of pathogens was determined by counting at least 50 cells per well, and the number of Gram-positive bacilli/cells (**c)** or *Candida albicans*/cells (**d**) were graphed in the histograms. Arrows indicate phagocytic cells. Data represent the mean ± SD of three independent experiments performed in duplicate. Different symbols indicate a significant difference according to ANOVA and Tukey’s test. *P < 0.05 vs. control group; ^&^*P* < 0.05 vs. LPS + IFN-γ-treated group

Phagocytosis is an important characteristic of macrophage activity. Compared with the M2-polarised or non-polarized BMDMs, M1-polarised BMDMs exhibited a higher phagocytic capacity for Gram-positive bacilli or fungi, no matter in the sum of phagocytic pathogens or the number of phagocytic cells (Fig. 4 LPS + IFN-γ-treated groups). After luteolin contribution, the phagocytic activity of M1-polarised macrophages was reduced slowly and showed a dose-effect relationship (Fig. 4 LPS + IFN-γ-combined with 2.5/5.0Lut-treated groups). In the mean time, it was found that non-polarized cells also have a certain phagocytic function (Fig. 4 control groups).

### Effect of luteolin on mRNA expression of inflammatory factors in polarised BMDMs

**Fig. 5:**
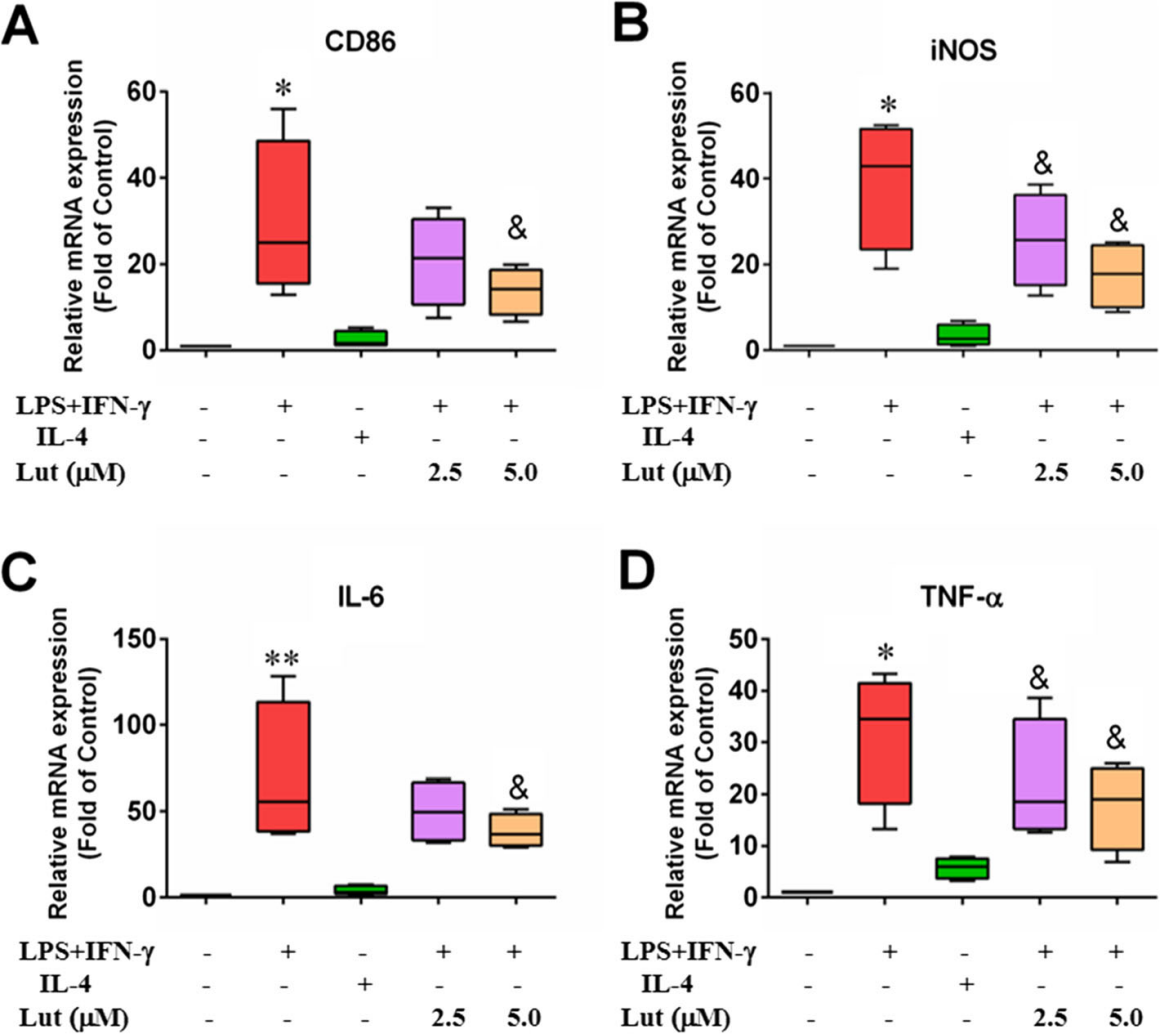
Effect of luteolin on the expression of M1-type pro-inflammatory factors in activated BMDMs. The M1-type mRNA molecules were determined by qPCR with GAPDH as an internal control. BMDMs were primed with LPS + IFN-γ or IL-4, and LPS + IFN-γ-treated-BMDMs were incubated with indicated doses of luteolin for 24 h. As a result, the relative M1-type mRNA levels of CD86 (**a**), iNOS (**b**), IL-6 (**c**) and TNF-α (**d**) in M1-polarised macrophages reduced slowly. Data represent the mean ± SD of four independent experiments performed in duplicate. Different symbols indicate a significant difference according to ANOVA and Tukey’s test. *P < 0.05, **P < 0.01 vs. control group (non-treated control); ^&^*P* < 0.05 vs. LPS + IFN-γ-treated group

**Fig. 6:**
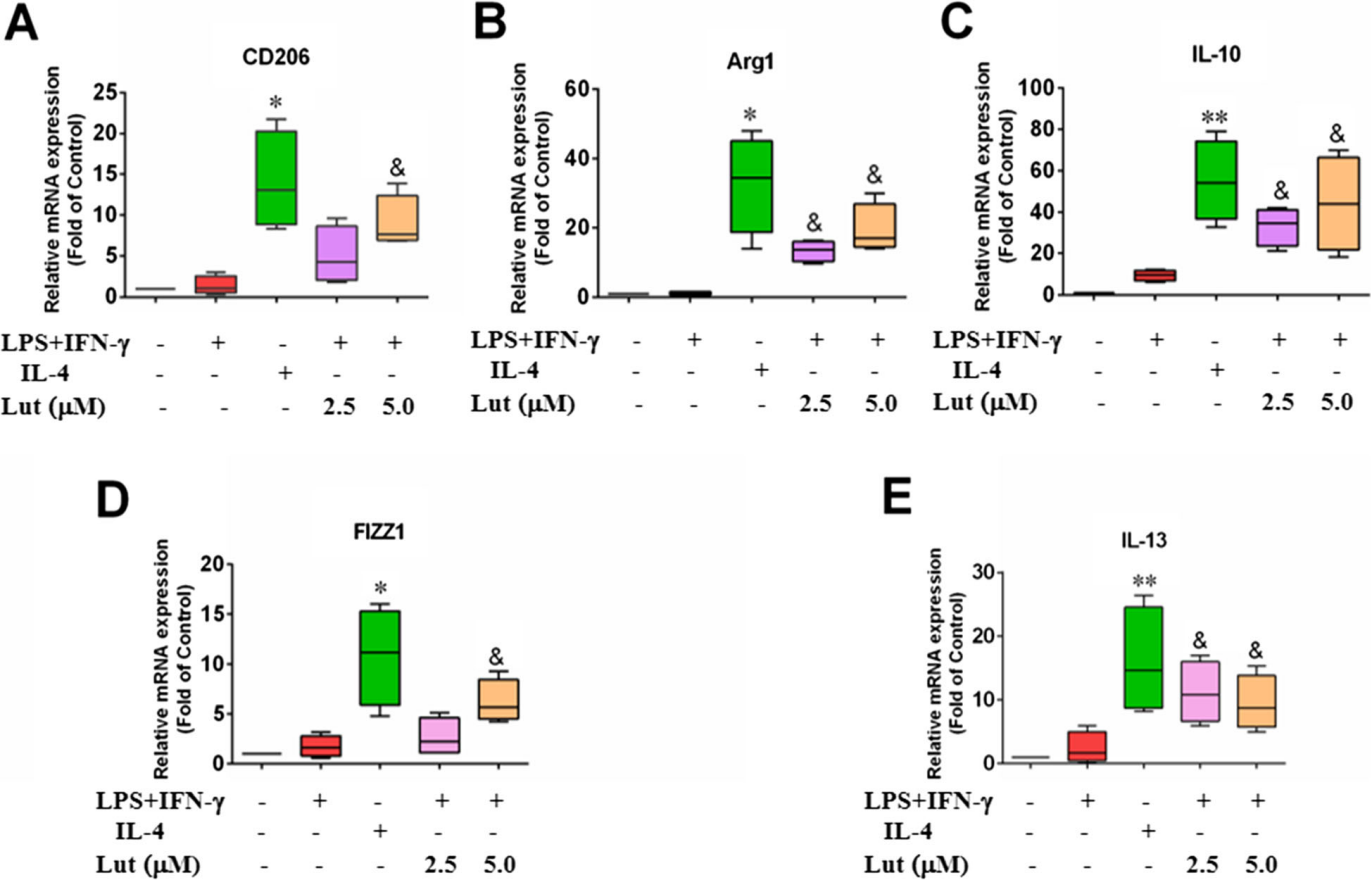
Effect of luteolin on the expression of M2-type anti-inflammatory factors in activated BMDMs. The M2-type mRNA molecules were assessed by qPCR with GAPDH as an internal control. BMDMs were primed with LPS + IFN-γ or IL-4, and LPS + IFN-γ-treated BMDMs incubated with the indicated doses of luteolin for 24 h, then the relative M2-type mRNA levels CD206 (**a**), Arg1 (**b**), IL-10 (**c**), FIZZ1 (**d**) and IL-13 (**e**) in M1-polarised macrophages elevated gradually. Data represent the mean ± SD of four independent experiments performed in duplicate. Different symbols indicate a significant difference according to ANOVA and Tukey’s test. *P < 0.05, **P < 0.01 vs. control group (without treatment); ^&^*P* < 0.05 vs. LPS + IFN-γ-treated group

The expression of M1-type and M2-type inflammatory factors was detected by qPCR. Results implied that the expression of M1-type pro-inflammatory factors in M1-polarised BMDMs such as CD86, iNOS, TNF-α and IL-6 was upregulated; the expression of M2 type anti-inflammatory factors such as CD206, Arg1, IL-10 FIZZ1 and IL-13 in M2-polarised BMDMs was also upmodulated. This indicates that the BMDM polarisation models were successfully induced. When M1 cells were combined with luteolin, the expression of M1-type pro-inflammatory factors were decreased in a dose-dependent pattern (Fig. 5), while the expression of M2-type anti-inflammatory factors was increased, but not all in a concentration-dependent manner (Fig. 6).

### Effect of luteolin on inflammatory cytokine levels in polarised BMDMs

**Fig. 7:**
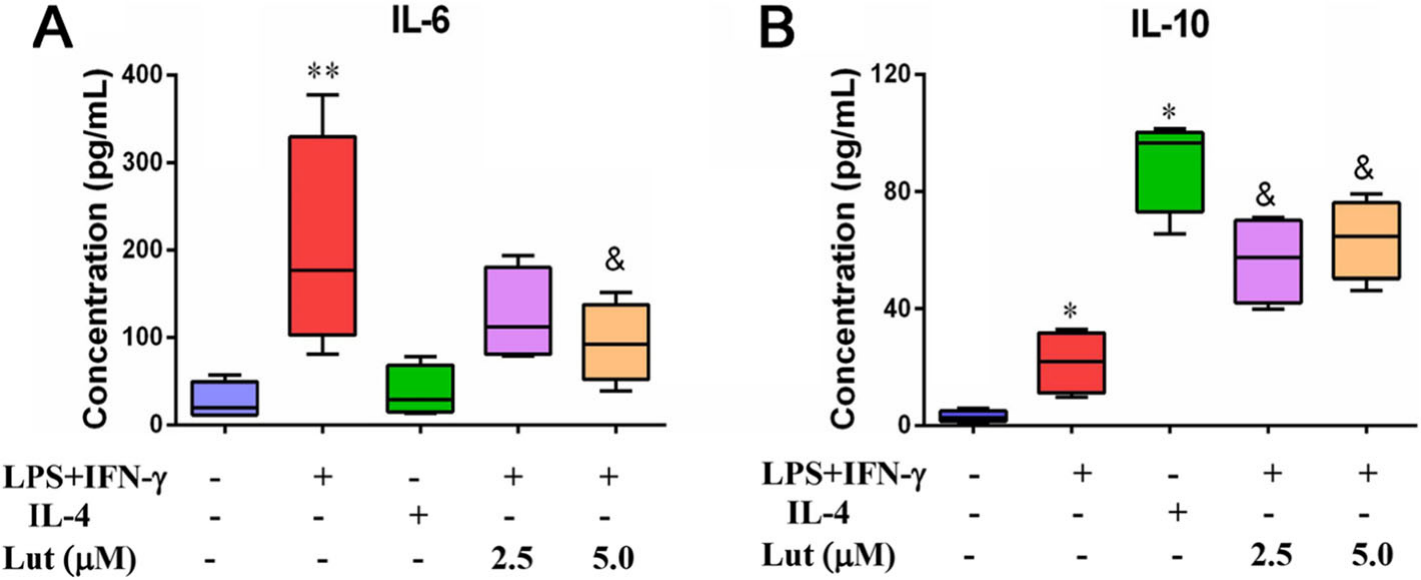
Effect of luteolin on IL-6 and IL-10 levels in polarised BMDMs. BMDMs were primed with LPS + IFN-γ or IL-4, followed by luteolin exposure for 24 h. Supernatants were harvested, and levels of IL-6 (**a**) and IL-10 (**b**) secreted from the M1-polarised BMDMs were measured via ELISA. Data represent the mean ± SD of four independent experiments performed in duplicate. Different symbols indicate a significant difference according to ANOVA and Tukey’s test. *P < 0.05 vs. control group (without treatment); ^&^*P* < 0.05 vs. LPS + IFN-γ-treated group

To further explore the polarity-skewing effect of luteolin in BMDMs, IL-6 and IL-10 production were measured by ELISA. The pro-inflammatory cytokine IL-6 liberated by M1-polarised BMDMs increased significantly, in the meantime, anti-inflammatory cytokine IL-10 secreted by M2-polarised BMDMs was also amplificated clearly, which was significantly different from the levels of the control group (without treatment). After various doses of luteolin were challenged to M1-polarized BMDMs, IL-6 released by M1-polarized BMDMs lowered visibly, while IL-10 elevated obviously, compared with those in the corresponding LPS + IFN-γ-treated group, with a statistical difference (Fig. 7).

### Effect of luteolin on the expression of surface markers on polarised BMDMs

**Fig. 8:**
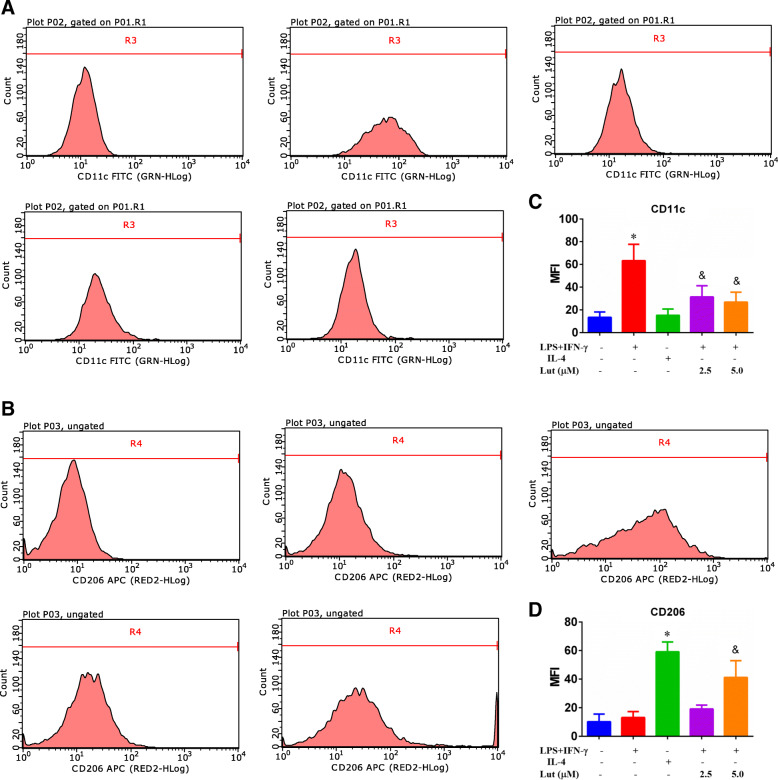
Effect of luteolin on the expression of BMDM surface markers CD11c and CD206. The BMDMs were stimulated with LPS + IFN-γ or IL-4, followed by luteolin treatment for 24 h. The expression levels of CD11c (**a**) and CD206 (**b**) protein on BMDMS are presented as MFI as evaluated by FCM. The histograms present the MFI of CD11c (**c**) and CD206 (**d**). Data represent the mean ± SD of three independent experiments. Different symbols indicate a significant difference according to ANOVA and Tukey’s test. *P < 0.05 vs. control group (without treatment); ^&^*P* < 0.05 vs. LPS + IFN-γ-treated group

CD11c and CD206 are the surface markers of M1-polarised or M2-polarised BMDMs, respectively. The FCM results implied that the MFI of CD11c (60.81) in M1-polarised BMDMs was significantly enhanced compared with that of the IL-4 treatment group (13.84) and control group (11.59). The MFI of CD206 (58.36) in M2-polarised BMDMs was also significantly amplified than that in the LPS + IFN-γ alone treatment group (12.46) and control group (7.33). Conversely, luteolin treatment dramatically attenuated the MFI of CD11c to 29.56 (LPS + IFN-γ + 2.5Lut-treated group) and 23.02 (LPS + IFN-γ + 5.0Lut-treated group) in M1-polarised BMDMs, but gradually strengthened the MFI of CD206 to 16.12 (LPS + IFN-γ + 2.5Lut-treated group) and 38.77 (LPS + IFN-γ + 5.0Lut-treated group) in M1-polarized BMDMs in a concentration-dependent model (Fig. 8).

### Effect of luteolin on protein pathway in polarised BMDMs

**Fig. 9:**
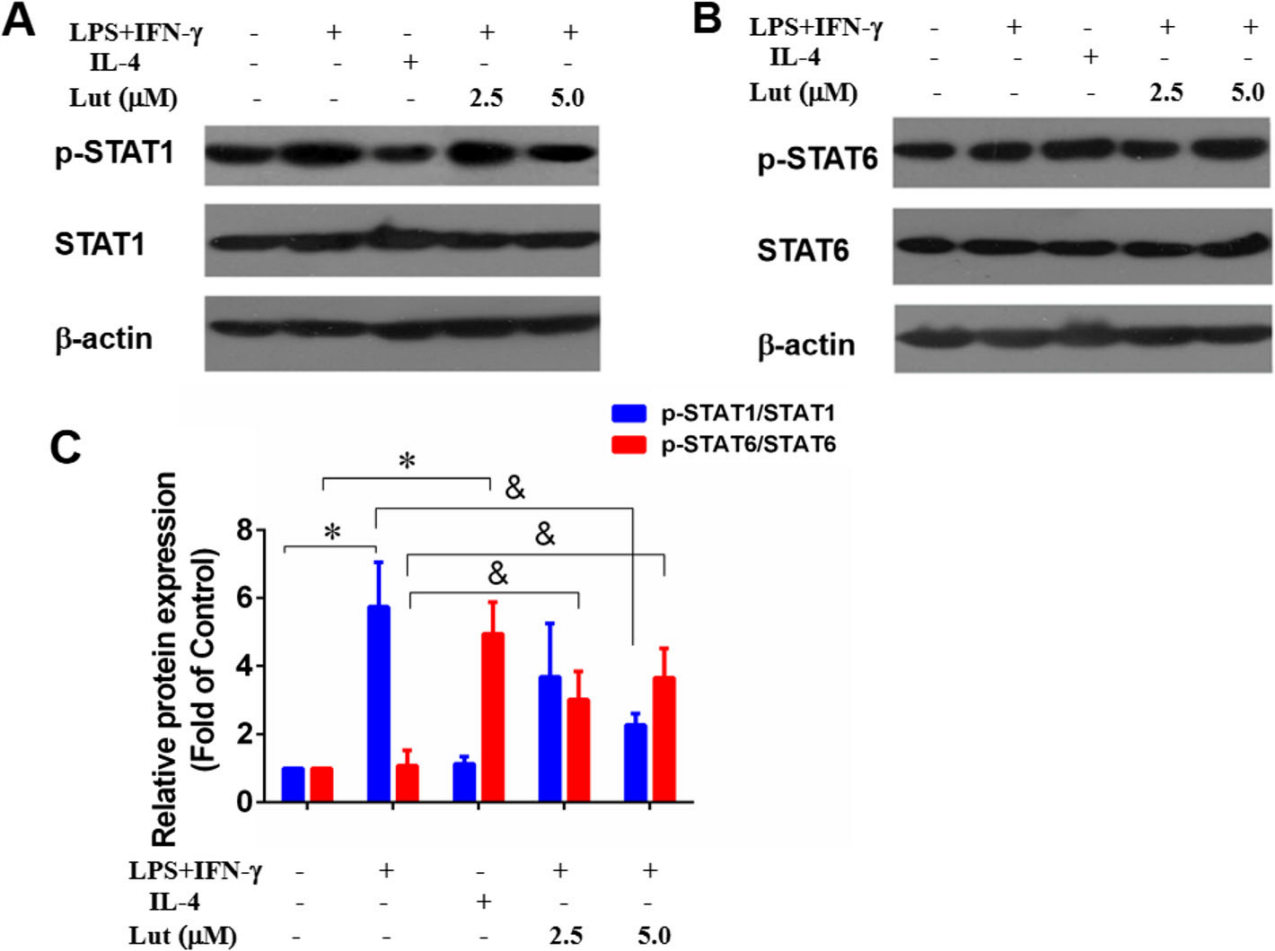
Effects of luteolin on the protein levels of p-STAT1/6 in polarised BMDMs. The BMDMs were primed with LPS + IFN-γ or IL-4, and then for addition of luteolin for 24 h. Total cell lysates were analysed by immunoblotting for the indicated antibody, and β-actin was used as the loading control. Representative immunoblots of p-STAT1 (**a**) and p-STAT6 (**b**); the relative protein levels of p-STAT1 and p-STAT6 (**c**) by densitometric analysis. Data represent the mean ± SD of three independent experiments. Different symbols indicate a significant difference according to ANOVA and Tukey’s test. *P < 0.05 vs. control group (without treatment); ^&^*P* < 0.05 vs. LPS + IFN-γ-treated group

STAT signaling proteins exert a vital role in macrophage polarisation and the expression of inflammatory cytokines in sepsis. Immunoblotting assay and densitometry analysis of STAT proteins revealed that M1-polarised BMDMs highly expressed p-STAT1 and lowly expressed p-STAT6, whereas M2-polarised BMDMs lowly expressed p-STAT1 and highly expressed p-STAT6. Predominantly, after luteolin contribution, p-STAT1 expression was depressed, while p-STAT6 was strengthened in the protein pathway of M1-polarised BMDMs (Fig. 9).

## Discussion

Infectious diseases are a leading cause of death, and the severity of infection is due to the exaggerated activation of macrophages and the “cytokine storm” [19]. LPS, the main component of endotoxin, is the outer membrane structure of the cell wall of Gram-negative bacteria, which can bind to toll-like receptor 4 on the macrophage surface to induce M1 polarisation and secrete pleiotropic cytokines [20]. IFN-γ can synergise with LPS to further activate cells, secrete excessive cytokines, cause SIRS, and in severe cases, result in sepsis and MOF. Therefore, it is particularly important to regulate macrophage polarisation, avoid excessive activation of M1 macrophages, reduce inflammation and promote tissue repair. BMDMs are suitable cell models for studying macrophage polarisation. In this investigation, bone marrow cells derived from femurs of C57BL/6 mice were stimulated to develop and differentiate into mature BMDMs using M-CSF. LPS and IFN-γ stimulated BMDMs to undergo M1 polarisation, the phagocytic activity was enhanced, and the M1-type pro-inflammatory factors including iNOS, TNF-α, IL-6, and surface markers CD86 and CD11C were upregulated. Meanwhile, IL-4 stimulated M2 polarisation in BMDMs, and M2-type anti-inflammatory factors including Arg1, IL-10, IL-13, FIZZ1 and CD206 were upregulated, indicating successful induction of M1/2 polarisation in BMDMs. After luteolin administration, the phagocytic activity or M1-type pro-inflammatory factors decreased and the M2-type anti-inflammatory factors increased evidently in M1-polarised BMDMs. Concurrently, expression of the p-STAT1 protein pathway was downregulated and p-STAT6 expression was upregulated. This suggests that luteolin may modulate the polarisation phenotype of BMDMs through the inhibition of p-STAT1 and the activation of p-STAT6, transforming it from the pro-inflammatory M1-type to the anti-inflammatory M2-type, thereby reducing the expression of inflammatory mediators and alleviating inflammation to maintain the stability of the microenvironment.

Cell polarisation is regulated by various signalling molecules and transcription factors. STATs are pivotal signal transduction pathways and widely involved in the process of cell activation, apoptosis, inflammation and immune regulation [21]. Studies by Sodhi et al. [22] and Zhou et al. [23] showed that IL-6 and IFN-γ released by LPS-polarised M1 macrophages can promote the expression of STAT1 protein, and that IFN-γ can also motivate STAT1 by binding to its receptor, and simultaneously, the activated STAT1 can further provoke the levels of TNF-α, IL-1β and iNOS in macrophages. iNOS is a signature of M1-polarised macrophages, which are responsible for nitric oxide (NO) production when cells are stimulated by IFN-γ or LPS, and NO is the essential condition for macrophages to perform phagocytic function, furthermore, excessive NO causes oxidative stress and inflammatory damage [24]. Similarly, phagocytosis can clear pathogens, but excessive phagocytosis will drive M1-polarisation of macrophages and an overactive immune response [25]. Both CD86 and CD11c are surface markers of M1 macrophages. CD86 is a B7 costimulatory molecule that stimulates the activation of antigen-presenting cells to secrete more pro-inflammatory factors. In the meantime, the level of CD86 can reflect and positively correlate with the level of cytokines such as IFN-γ and IL-12, while IL-10 can hinder the level of CD86 [26, 27]. CD11c is often coupled with CD18 and binds to bacterial LPS, which activates CD4^+^T cells to proliferate and differentiate into Th1 cells and secrete massive TNF-α, IL-6 and IL-12 to trigger inflammatory cascades [28]. Two other crucial cytokines, IL-6 and TNF-α, which generated in abundance by IL-1β stimulation or autocrined from the activated “mononuclear-macrophage system”, are elevated not only in bacterial infection but also during viral infection [29]. More importantly, they are the strongest pro-inflammatory agents causing a “cytokine storm”. Studies have shown that patients with severe COVID-19 characterised by a “cytokine storm” inexorably exhibit high levels of IL-6 and TNF-α in serum, and IL-6 or TNF-α antagonist seem to be very promising for severe COVID-19 cases [30].

To our knowledge, IL-4 or IL-13 can induce M2-type polarisation of macrophages, and M2-type anti-inflammatory factors such as IL-10, IL-13, FIZZ1, Arg1 and CD206 are upmodulated [31]. In this regard, IL-4 or IL-13 binds to its receptor to activate JAK to further phosphorylate STAT6 and enhance the Arg1 activity. Arg1 and iNOS are important hallmarks of M2/M1-type macrophage polarisation, respectively. Under normal circumstances, the activities of Arg1 and iNOS are strictly regulated by macrophages and maintain a dynamic equilibrium. When M2 polarisation occurs, Arg1 competes for iNOS to decompose the substrate arginine, thus benefits for tissue regeneration [24]. Moreover, Arg1 is also inseparable from M2 macrophage properties in playing immune memory function to eliminate infectious agents. CD206, so called mannose receptor, is a membrane surface marker of M2 cells, which can specifically recognise antigens to clear pathogens, promote angiogenesis and repress the immune response [32]. FIZZ1 is also known as resistin-like molecule α, which participates in killing bacteria, viruses and other pathogens by regulating cellular immunity and promotes angiogenesis, tissue repair and wound healing [33]. Another M2-type anti-inflammatory factor, IL-10, on the one hand, enhances the sensitivity of macrophages to IL-4 and IL-13 by increasing the abundance of IL-4 receptors on the macrophage surface, which contributes to M2-type polarisation of macrophages. On the other hand, it can synergise with IL-4 to inhibit pro-inflammatory cytokines IL-1β and TNF-α to reduce inflammation. In light of preliminary data, IL-10 displayed higher levels in patients with sepsis and serious COVID-19. All these indicate that when inflammation is motivated, an intricate network is formed between the pro-inflammatory mediators and activated STAT1, eliciting an “inflammatory storm.” Nevertheless, upon luteolin administration, a complex network is also formed between anti-inflammatory mediators and activated STAT6, further facilitating the expression of anti-inflammatory factors, which resist the formation of pro-inflammatory factors and alleviate inflammation accordingly. The herbal compound physalin D can repolarise M1 toward M2 polarisation in BMDMs through STAT1 suppression and STAT6 activation [34], which is consistent with our study.

## Conclusion

Altogether, the BMDM polarisation mechanism is complex and involves many protein pathways. Only by actively exploring the regulation of BMDM polarisation and maintaining the balance of inflammatory mediators can maintain the physical stable. In this investigation, LPS/IFN-γ induced M1 polarisation and IL-4 induced M2 polarisation of BMDMs. After treatment with herbal compound luteolin, the M1-polarised BMDMs showed lowered levels of M1-type pro-inflammatory factors and elevated levels of M2-type anti-inflammatory factors, and that the signalling protein p-STAT1 was downregulated and p-STAT6 was upregulated. That is, the BMDM population underwent a transformation from a pro-inflammatory M1-phenotype to an anti-inflammatory M2-phenotype. Simultaneously, inflammatory factors are analogously altered from pro-inflammatory to anti-inflammatory type. In light of these findings, our research provides a novel insight into the role of plant-derived luteolin to be a candidate for controlling the macrophage phenotype to treat infectious diseases.

## Data Availability

All data generated or analysed during this study are included in this published article and are available from the corresponding author upon request.

## Abbreviations

ARDS: Acute respiratory distress syndrome
Arg: Arginase
BMDMs: Bone marrow-derived macrophages
CD: Cluster of differentiation
FIZZ: Found in inflammatory zone
IFN: Interferon
IL: Interleukin
iNOS: Inducible nitric oxide synthase
MOF: Multiple-organ failure
LPS: Lipopolysaccharide
NO: Nitric oxide
SIRS: Systemic inflammatory response syndrome
STAT: Signal transducer and activator of transcription
TNF: Tumour necrosis factor

## References

[1] Chousterman BG, Swirski FK, Weber GF (2017). Cytokine storm and sepsis disease pathogenesis. Semin Immunopathol.

[2] Greten FR, Grivennikov SI (2019). Inflammation and cancer: triggers, mechanisms, and consequences. Immunity..

[3] Peter AE, Sandeep BV, Rao BG, Kalpana VL (2021). Calming the storm: natural Immunosuppressants as adjuvants to target the cytokine storm in COVID-19. Front Pharmacol.

[4] Li H, Liu L, Zhang DY, Xu JY, Dai HP, Tang N (2020). SARS-CoV-2 and viral sepsis: observations and hypotheses. Lancet..

[5] Liu Q, Zhou YH, Yang ZQ (2016). The cytokine storm of severe influenza and development of immunomodulatory therapy. Cell Mol Immunol.

[6] Nurrahmah QI, Madhyastha R, Madhyastha H, Purbasari B, Maruyama M, Nakajima Y (2021). Retinoic acid abrogates LPS-induced inflammatory response via negative regulation of NF-kappa B/miR-21 signaling. Immunopharmacol Immunotoxicol.

[7] Kohno K, Koya-Miyata S, Harashima A, Tsukuda T, Katakami M, Ariyasu T (2021). Inflammatory M1-like macrophages polarized by NK-4 undergo enhanced phenotypic switching to an anti-inflammatory M2-like phenotype upon co-culture with apoptotic cells. J Inflamm (Lond).

[8] Amano MT, Castoldi A, Andrade-Oliveira V, Latancia MT, Terra FF, Correa-Costa M (2018). The lack of PI3K favors M1 macrophage polarization and does not prevent kidney diseases progression. Int Immunopharmacol.

[9] De Campos GY, Oliveira RA, Oliveira-Brito PKM, Roque-Barreira MC, da Silva TA (2020). Pro-inflammatory response ensured by LPS and Pam3CSK4 in RAW 264.7 cells did not improve a fungistatic effect on Cryptococcus gattii infection. PeerJ.

[10] Podd BS, Simon DW, Lopez S, Nowalk A, Carcillo JA, Aneja R (2017). Rationale for adjunctive therapies for pediatric sepsis induced multiple organ failure. Pediatr Clin N Am.

[11] Wong TY, Tan YQ, Lin SM, Leung LK (2017). Apigenin and luteolin display differential hypocholesterolemic mechanisms in mice fed a high-fat diet. Biomed Pharmacother.

[12] Raina R, Pramodh S, Rais N, Haque S, Shafarin J, Bajbouj K (2021). Luteolin inhibits proliferation, triggers apoptosis and modulates Akt/mTOR and MAP kinase pathways in HeLa cells. Oncol Lett.

[13] Kim SH, Saba E, Kim BK, Yang WK, Park YC, Shin HJ (2018). Luteolin attenuates airway inflammation by inducing the transition of CD4(+)CD25(−) to CD4(+)CD25(+) regulatory T cells. Eur J Pharmacol.

[14] Yan H, Ma L, Wang H, Wu S, Huang H, Gu Z (2019). Luteolin decreases the yield of influenza a virus in vitro by interfering with the coat protein I complex expression. J Nat Med.

[15] Peng M, Watanabe S, Chan KWK, He Q, Zhao Y, Zhang Z (2017). Luteolin restricts dengue virus replication through inhibition of the proprotein convertase furin. Antivir Res.

[16] Huang YF, Bai C, He F, Xie Y, Zhou H (2020). Review on the potential action mechanisms of Chinese medicines in treating coronavirus disease 2019 (COVID-19). Pharmacol Res.

[17] Saeedi-Boroujeni A, Mahmoudian-Sani M-R (2021). Anti-inflammatory potential of Quercetin in COVID-19 treatment. J Inflamm (Lond).

[18] Wang SX, Cao M, Xu SH, Zhang JM, Wang ZG, Mao XD (2017). Effect of luteolin on inflammatory responses in RAW264.7 macrophages activated with LPS and IFN-γ. J Funct Foods.

[19] Srikiatkhachorn A, Mathew A, Rothman AL (2017). Immune-mediated cytokine storm and its role in severe dengue. Semin Immunopathol.

[20] Pedraza-Sánchez S, Vargas-Hernmmune-mediated cytokine storm and its role in severe dengueentella A (2020). THP-1 cells increase TNF-alpha production upon LPS + soluble human IgG co-stimulation supporting evidence for TLR4 and Fcgamma receptors crosstalk. Cell Immunol.

[21] Alhetheel A, Yakubtsov Y, Abdkader K, Sant N, Diaz-Mitoma F, Kumar A (2008). Amplification of the signal transducer and activator of transcription I signaling pathway and its association with apoptosis in monocytes from HIV-infected patients. AIDS..

[22] Sodhi A, Kesherwani V. Signaling molecules involved in production and regulation of IL-1beta by murine peritoneal macrophages in vitro on treatment with concanavalin a. Int Immunopharmacol. 2007;7:1403–13. 10.1016/j.intimp.2007.07.004 17761344

[23] Zhou DX, Huang C, Lin Z, Zhan SX, Kong LN, Fang CB (2014). Macrophage polarization and function with emphasis on the evolving roles of coordinated regulation of cellular signaling pathways. Cell Signal.

[24] Rath M, Müller I, Kropf P, Closs EI, Munder M (2014). Metabolism via arginase or nitric oxidesynthase: two competing arginine pathways in macrophages. Front Immunol.

[25] Deng H, Li ZC, Tan YF, Guo ZB, Liu YY, Wang Y (2016). A novel strain of Bacteroides fragilis enhances phagocytosis and polarises M1 macrophages. Sci Rep.

[26] Philipp D, Suhr L, Wahlers T, Choi YH, Paunel-Görgülü A (2018). Preconditioning of bone marrow-derived mesenchymal stem cells highly strengthens their potential to promote IL-6-dependent M2b polarization. Stem Cell Res Ther.

[27] Soltys J, Bonfield T, Chmiel J, Berger M (2002). Functional IL-10 deficiency in the lung of cystic fibrosis (cftr(−/−)) and IL-10 knockout mice causes increased expression and function of B7 costimulatory molecules on alveolar macrophages. J Immunol.

[28] Sándor N, Lukácsi S, Ungai-Salánki R, Orgován N, Szabó B, Horváth R (2016). CD11c/CD18 dominates adhesion of human monocytes, macrophages and dendritic cells over CD11b/CD18. PLoS One.

[29] Indalao IL, Sawabuchi T, Takahashi E, Kido H (2017). IL-1β is a key cytokine that induces trypsin upregulation in the influenza virus-cytokine-trypsin cycle. Arch Virol.

[30] Feldmann M, Maini RN, Woody JN, Holgate ST, Winter G, Rowland M (2020). Trials of anti-tumour necrosis factor therapy for COVID-19 are urgently needed. Lancet..

[31] Duan JX, Zhou Y, Zhou AY, Guan XX, Liu T, Yang HH (2017). Calcitonin gene-related peptide exerts anti-inflammatory property through regulating murine macrophages polarization in vitro. Mol Immunol.

[32] Dai K, Huang L, Sun XM, Yang LH, Gong ZJ (2015). Hepatic CD206-positive macrophages express amphiregulin to promote the immunosuppressive activity of regulatory T cells in HBV infection. J Leukoc Biol.

[33] Choe SH, Choi EY, Hyeon JY, Keum BR, Choi IS, Kim SJ (2021). Effect of nifedipine, a calciumchannel blocker, on the generation of nitric oxide and interleukin-1β by murine macrophages activated by lipopolysaccharide from Prevotella intermedia. Naunyn Schmiedeberg's Arch Pharmacol.

[34] Ding N, Wang YX, Dou C, Liu FL (2019). GuanG, Wei KY, et al. Physalin D regulates macrophage M1/M2 polarization via the STAT1/6 pathway. J Cell Physiol.

